# Socket Shield Technique in Implant Dentistry: A Systematic Review and Meta-Analysis of Survival, Aesthetic Outcomes, and Complications

**DOI:** 10.7759/cureus.85176

**Published:** 2025-06-01

**Authors:** Abdulaziz M Altalhi, Hussain H AlHajji, Sahar K Alharbi, Abrar R Alanzi, Zaizafon M Baslom, Jehan A Omar, Layan A AlShenefi

**Affiliations:** 1 Dentistry, Ministry of Health, Riyadh, SAU; 2 Dentistry, Ministry of Health, Dammam, SAU; 3 Dentistry, Raseel Medical Center, Riyadh, SAU; 4 Dentistry, Umm Al-Qura University, Mecca, SAU; 5 Dentistry, Ministry of Health, Jeddah, SAU; 6 Dentistry, Majmaah University, Al Majma'ah, SAU

**Keywords:** aesthetic outcomes, dental implants, implant survival rate, peri-implant tissue preservation, socket shield technique

## Abstract

The socket shield technique (SST) is an innovative approach in implant dentistry designed to preserve the buccal bone plate and soft tissue aesthetics during implant placement. By retaining a portion of the tooth root, the SST aims to reduce alveolar ridge resorption and improve peri-implant tissue stability. This systematic review evaluates the survival outcomes and limitations of the SST through an analysis of longitudinal studies published between January 2019 and December 2024. A comprehensive search of PubMed/Medline, Scopus, and Google Scholar identified 448 articles, of which 11 met the inclusion criteria. Several key outcomes were assessed, including implant survival rates, Pink Esthetic Scores, marginal bone loss, and complications, such as shield exposure and peri-implantitis. The findings demonstrate that the SST reduces bone resorption and enhances aesthetic outcomes, particularly in the anterior region. However, challenges such as technical sensitivity, shield mobility, and limited long-term data limit its applicability in clinical settings. Additionally, variations in methodology among studies underscore the need for further high-quality research. This review highlights the importance of meticulous surgical execution, patient selection, and standardized protocols to optimize SST outcomes. Future directions should prioritize long-term evaluations, advanced digital workflows, and innovative biomaterials to address current limitations and enhance the clinical utility of the SST in implant dentistry.

## Introduction and background

Significant advancements have been made in implant dentistry over the years, with the primary goal of restoring both function and aesthetics while minimizing biological and mechanical complications [1]. Preserving alveolar bone and soft tissue during implant placement remains a critical factor in ensuring long-term success and patient satisfaction. Among various techniques proposed to address these challenges, such as socket preservation grafting, immediate implant placement, and guided bone regeneration, the socket shield technique (SST) has garnered increasing interest due to its potential to preserve the buccal bone plate, minimize tissue collapse, and improve aesthetic outcomes [2].

Tooth extraction often results in alveolar ridge resorption, which primarily affects the buccal plate [3]. This bone loss can compromise the implant site, necessitating additional grafting procedures or affecting the aesthetics and function of the final restoration [4]. Conventional approaches, such as immediate implant placement, have been successful in mitigating bone loss; however, achieving predictable outcomes remains a challenge due to biological remodeling [5]. The SST, first described by Hürzeler et al. (2010), offers an innovative solution by retaining a partial segment of the root (i.e., the buccal shield) during implant placement [6]. This approach aims to maintain the periodontal ligament and bundle bone complex, which are otherwise lost post-extraction.

The SST involves the deliberate retention of the buccal portion of the root, while the remaining tooth is carefully extracted. The preserved buccal shield serves as a scaffold for soft tissue and bone stability, preventing the resorption of the buccal plate [7]. In addition to aesthetic benefits, the SST has been associated with improved implant survival rates and reduced marginal bone loss [8].

Despite its reported advantages, there are several limitations to the adoption of the SST in clinical practice, including technical sensitivity, limited longitudinal data, and potential complications such as root fragment infection [9,10]. While initial studies have demonstrated promising outcomes, systematic evaluation of survival rates, patient satisfaction, and associated complications is necessary to establish standardized protocols and provide evidence-based recommendations for clinicians [11,12].

This systematic review aims to synthesize existing evidence on the survival outcomes and limitations of the SST based on longitudinal studies. By analyzing studies published from January 2019 to December 2024, it provides a comprehensive assessment of the technique’s clinical efficacy and challenges. The findings aim to guide clinicians in applying the SST appropriately and inform future research directions to address current knowledge gaps.

## Review

Methods

Review Protocol

The present review adhered to the Preferred Reporting Items for Systematic reviews and Meta-Analyses (PRISMA) guidelines to ensure a rigorous and transparent methodology. A structured approach based on the Population, Intervention, Comparison, Outcomes, and Study (PICOS) framework was employed to define the research protocol. These were as follows: Population (P): Human participants who underwent immediate implant placement with or without the SST. Intervention (I): The application of the SST during implant placement to preserve the buccal bone plate and peri-implant soft tissue. Comparison (C): Conventional immediate implant placement techniques that do not involve partial root retention. Outcome (O): Evaluation of implant survival rates, Pink Esthetic Scores (PES), marginal bone loss, and complications (e.g., shield exposure, peri-implantitis). Study Design (S): This review included randomized controlled trials (RCTs), prospective cohort studies, case-control studies, and case series that focused on the SST.

The review aimed to assess the clinical efficacy and survival outcomes of the SST compared to conventional implant placement techniques. Additionally, it sought to evaluate complications, patient satisfaction, and aesthetic outcomes associated with the SST.

Studies included in this review specifically addressed survival outcomes and limitations of the SST in human participants and involved various age groups and clinical settings. The exclusion criteria were studies that did not explicitly compare SST with other techniques, in vitro or animal studies, editorials, narrative reviews, conference abstracts, and articles published in languages other than English. By applying these criteria, the review aimed to provide a comprehensive synthesis of the evidence supporting the efficacy and limitations of the SST in implant dentistry.

Database Search Strategy

The database search strategy for this systematic review comprised the use of three major databases to ensure comprehensive coverage of relevant studies. The search strategy was tailored to the specific indexing systems and conventions of each platform. The objective was to identify all relevant studies on the SST, focusing on survival outcomes, complications, and long-term efficacy. Search strings were developed using Boolean operators, MeSH terms, and database-specific filters.

Table 1 presents the database-specific search strategies used to identify studies on the SST. Searches were performed in PubMed/MEDLINE, Scopus, and Google Scholar using combinations of MeSH terms, keywords, and Boolean operators to ensure comprehensive coverage of literature related to implant survival, aesthetic outcomes, and associated complications.

**Table 1:** Database-specific search strategy for identifying studies on the SST MEDLINE, Medical Literature Analysis and Retrieval System Online; MeSH, Medical Subject Headings; SST, socket shield technique

Database	Search string
PubMed/Medline	"Socket Shield Technique" [MeSH Terms] AND ("dental implants" [MeSH Terms] OR "implantology") AND ("clinical outcomes" OR "treatment success")
Scopus	TITLE-ABS-KEY ("Socket Shield Technique") AND TITLE-ABS-KEY ("dental implants") AND ("clinical outcomes" OR "survival rate")
Google Scholar	"Socket Shield Technique" AND "dental implants" AND ("clinical outcomes" OR "success rates")

Data Extraction

The data extraction process for this systematic review used a structured approach to ensure the comprehensive and accurate collection of relevant information from the included studies. The primary aim was to gather detailed data on the survival outcomes, complications, and limitations associated with the SST to facilitate in-depth analysis and synthesis of the findings.

Key study characteristics were extracted, including the author(s), year of publication, study design, and the region or country where the study was conducted. Clinical details were also systematically collected, such as sample size, intervention methods (SST protocol), comparator techniques, and follow-up durations.

The outcome measures analyzed include implant survival rates, marginal bone loss, PES, and reported complications, such as shield exposure, peri-implantitis, or soft tissue recession. Specific technical information, including surgical protocols and the use of adjunctive technologies like digital workflows or computer-aided design/computer-aided manufacturing (CAD/CAM) tools, was also recorded. Additionally, the limitations of each study, such as sample size constraints, follow-up periods, and methodology-related challenges, were noted.

This detailed data extraction strategy ensured that all relevant findings were included to comprehensively evaluate the efficacy, benefits, and limitations of the SST in implant dentistry.

Bias Assessment

The Newcastle-Ottawa Scale (NOS) was used to evaluate the quality and methodological rigor of the included studies. It assesses three main domains: selection of study groups, comparability of groups, and ascertainment of the outcome of interest. Each domain includes several items that are scored to determine the overall risk of bias. For each item, the study was given a specific number of stars (ranging from 0 to 9) based on the level of risk of bias. A higher number of stars indicates a lower risk of bias. The scores from the three domains were then summed to obtain the overall quality assessment of the study.

Statistical Analysis

The computer program Review Manager (RevMan) Version 5.4 (The Cochrane Collaboration 2020) was used for the quantitative assessment of the studies included. Forest plots were used to visualize the results of the meta-analysis. The random effect model was chosen based on the assumption of heterogeneity in the included studies.

Results

**Figure 1:**
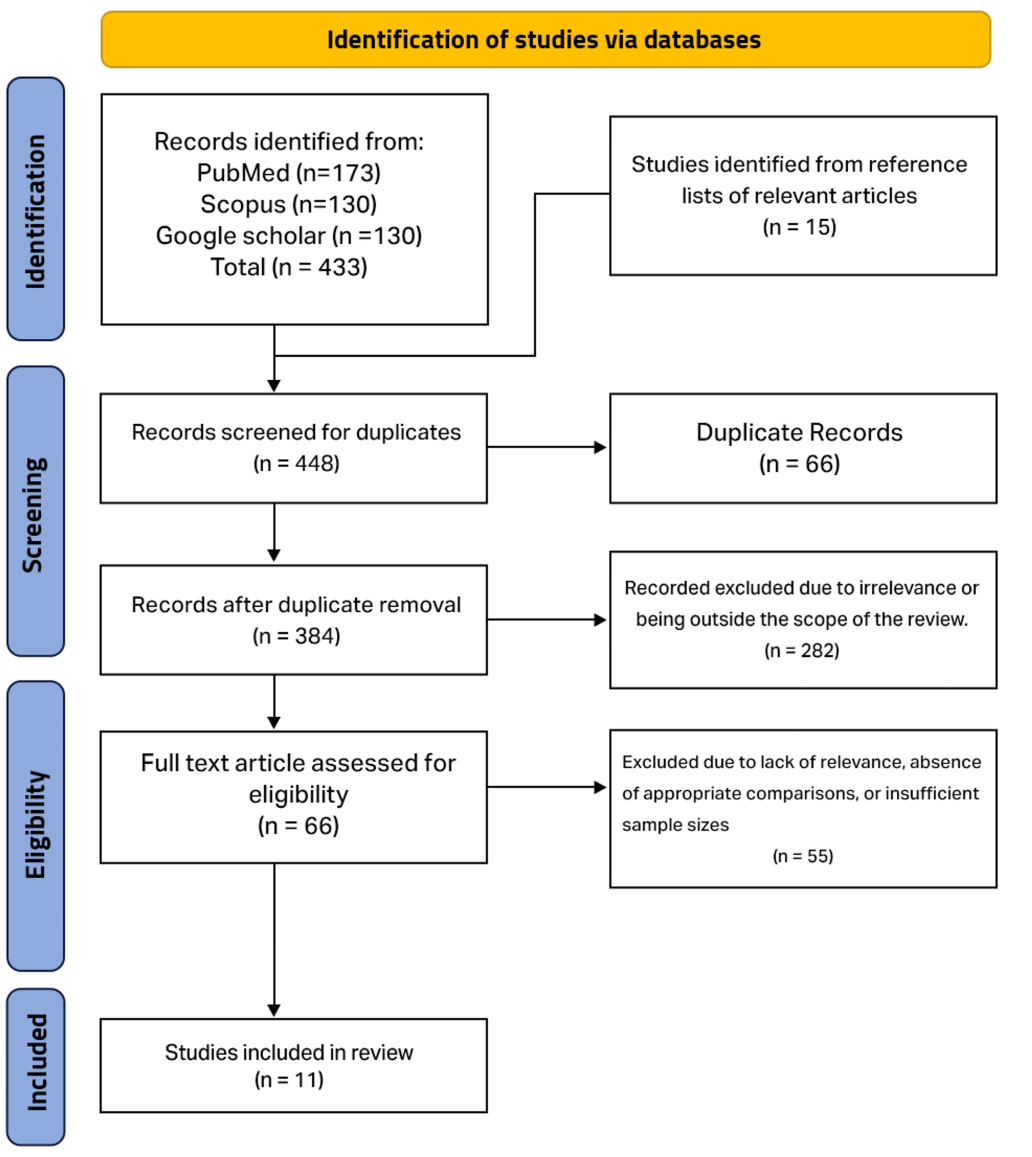
Flowchart summarizing the bibliographic search and the selection of articles

The initial stage of the review process identified 448 articles that were potentially relevant to the research question. A thorough screening process was then applied, beginning with the removal of duplicate articles, and it resulted in 384 unique articles. These articles underwent an initial screening based on their titles and abstracts, with those deemed irrelevant or outside the scope of the review excluded. Following this, 66 articles were selected for full-text review, during which they were assessed against predefined inclusion and exclusion criteria. Articles that were not relevant, lacked appropriate comparisons, or had insufficient sample sizes were excluded. After this evaluation, 11 studies were deemed suitable for inclusion in the review, as illustrated in Figure 1. This comprehensive process ensured that the final selection provided robust data on the survival outcomes and limitations of the SST.

Table 2 shows a summary of the included studies, highlighting their study design, sample size, key survival outcomes, and reported limitations associated with the SST. This comparative overview provides insight into the clinical performance and methodological quality of each study.

**Table 2:** Summary of included studies: survival outcomes and limitations of the SST DIP, delayed implant placement; DZ, demineralized zone; IIP, immediate implant placement; PES, Pink Esthetic Score; SST, socket shield technique

Study no.	Study reference	Study design	Sample size	Survival outcomes	Limitations
1	Atef et al. (2021) [13]	Randomized clinical trial	42 patients	SST demonstrated significantly less vertical (0.36 ± 0.62 mm) and horizontal (0.29 ± 0.34 mm) bone loss. PES was comparable in the SST (12.12 ± 0.64) and control (11.86 ± 0.35) groups. 100% implant success rate.	Technique sensitivity; lack of long-term data to validate findings
2	Esteve-Pardo and Esteve-Colomina (2023) [14]	Prospective case series	10 patients	Buccal bone shrinkage minimized (-0.22 ± 0.13 mm); volume loss reduction	Standardization of digital measurement methods is needed.
3	Gómez-Meda et al. (2022) [15]	Prospective cohort	26 interventions	Significant buccal bone thickness increase (0.65 ± 1.16 mm at 5 mm)	Small sample size; no aesthetic analysis performed
4	Habashneh et al. (2019) [16]	Case report series	Five cases	Stable esthetics and ridge preservation with successful functional outcomes at one-year follow-up	Short follow-up period; anecdotal evidence lacking robust controls
5	Pohl (2024) [17]	Case series	Six patients	Inter-implant papilla preservation between adjacent implants with favorable midterm outcomes	Limited to three- to five-year follow-up; requires validation through broader studies
6	Santhanakrishnan et al. (2021) [18]	Randomized trial	75 implants	Minimal reduction in crestal bone thickness in the SST group; superior PES compared to others	Limited statistical power; long-term results unclear
7	Santhanakrishnan et al. (2024) [19]	Randomized trial	75 patients	The SST group had lower crestal bone thickness reduction (0.09 mm) vs. IIP and DIP; PES was higher in SST.	High technical sensitivity; variations in patient factors not addressed
8	Shadid (2022) [20]	Prospective cohort	20 implants	Ridge width gain of 0.2 mm in the SST group; 100% osseointegration; better ridge preservation compared to DZ	Minor complications in two cases (external shield exposure); limited sample size
9	Shadid (2022) [21]	Case series	Five cases	Delayed implants showed stable facial bone and aesthetic outcomes with minimal marginal bone loss.	Lack of long-term evidence; larger trials are needed to generalize findings
10	Shadid (2022) [22]	Prospective case series	10 implants	Ridge width gain of 0.17 mm; 100% implant success rate; minimal bone loss (0.08-0.21 mm)	Limited follow-up (12 months); complications managed but noteworthy (e.g., external shield exposure)
11	Sun et al. (2020) [23]	Randomized clinical trial	30 patients	SST preserved the midfacial mucosa and buccal plate dimensions better than the control group. PES remained consistent (12.07 ± 1.62 in SST vs. 11.33 ± 1.76 in control) over 24 months.	Lack of significant PES difference between groups; absence of comparison of aesthetic satisfaction at the prosthetic level.

As the included studies were conducted across various geographic regions, the review provides a diverse representation of populations from different geographic and cultural contexts. This diversity enhances the applicability of the findings across various clinical settings. The sample sizes in the studies ranged from as small as five cases to as large as 75 patients, resulting in a mix of focused case-specific insights and broader population-level analyses. The participants were of a wide range of ages, although most studies did not specify detailed age demographics. The reported outcomes spanned from aesthetic evaluations, such as the PES, to survival rates and bone loss measurements, providing a comprehensive understanding of the SST’s efficacy and limitations in diverse clinical scenarios. The studies also varied in their methodologies, from randomized controlled trials to case series, contributing to a nuanced exploration of SST outcomes.

Table 3 provides a comprehensive summary of the 11 studies assessing the risk factors and complications associated with the SST.

**Table 3:** Risk factors and complications associated with the SST SST, socket shield technique

Risk factor/complication	Description	Frequency (%)	Reported studies
Shield exposure	Partial or complete exposure of the socket shield during healing or follow-up periods	10-20%	Atef et al. (2021) [13]; Shadid (2022) [20]
Shield mobility	Lack of proper integration or stability of the retained shield, leading to mobility	Not consistently reported (<5%)	Gómez-Meda et al. (2022) [15]; Shadid (2022) [21]
Shield fracture	Fracture of the socket shield during preparation or follow-up	Not consistently reported	Shadid (2022) [22]; Sun et al. (2020) [23]
Peri-implantitis	Inflammatory reactions affecting the peri-implant tissue due to plaque accumulation or exposure	5-10%	Esteve-Pardo and Esteve-Colomina (2023) [14]; Santhanakrishnan et al. (2024) [19]
Buccal bone loss	Unexpected or significant resorption of buccal bone post-implantation	5-15%	Gómez-Meda et al. (2022) [15]; Pohl (2024) [17]
Soft tissue recession	Marginal recession of the peri-implant soft tissues leading to aesthetic challenges	10-25%	Santhanakrishnan et al. (2021) [18]; Sun et al. (2020) [23]
Technical sensitivity	Errors related to preparation and execution due to the procedure’s high technical demands	Not quantifiable	Atef et al. (2021) [13]; Habashneh et al. (2019) [16]
Shield infection	Infection due to retained root fragments that were not fully cleaned during preparation	2-5%	Gómez-Meda et al. (2022) [15]; Shadid (2022) [22]
Limited evidence	Lack of sufficient long-term data to validate effectiveness and safety	Affects clinical confidence broadly	All studies

Main Findings

Qualitative assessment of the included studies demonstrated that the SST effectively preserved buccal bone and peri-implant soft tissue, resulting in reduced resorption and improved aesthetic outcomes. This indicates that SST can be a viable alternative to conventional implant placement techniques, especially in areas where aesthetics is of high importance. However, the studies highlighted the need for technical precision and standardized protocols to mitigate complications, such as shield exposure and peri-implantitis.

**Figure 2:**
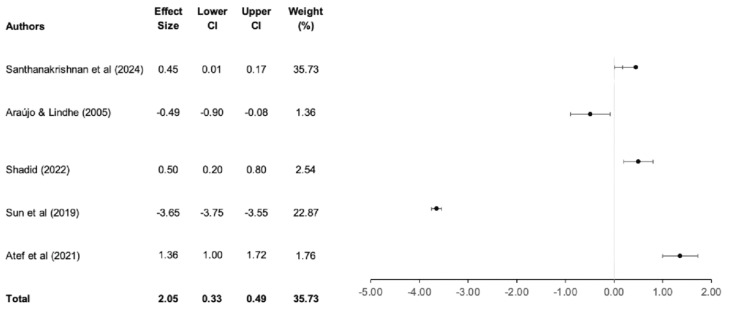
Forest plot comparing survival outcomes of the SST and conventional implant placements SST, socket shield technique Araújo and Lindhe (2005) [3]; Atef et al. (2021) [13]; Santhanakrishnan et al. (2024) [19]; Shadid (2022) [20]; Sun et al. (2020) [23]

Figure 2 shows a forest plot comparing the survival outcomes of implants placed using the SST versus conventional implant placement. The meta-analysis was conducted using a random-effects model, with results expressed as mean differences and 95% CIs.

A comparison of the survival outcomes and complications associated with the SST and conventional implant placement was analyzed using Review Manager version 5.4 at a 95% CI, as presented in Figure 2.

A total of 259 implants placed with SST were evaluated in the SST category compared to 355 in the conventional implant placement category. Among the five included studies, no significant differences in survival outcomes were observed at p = 0.08. A mean difference of 0.45 (95% CI: 0.01; 0.17) suggests that SST offers comparable survival outcomes to traditional methods while providing enhanced aesthetic benefits. Statistical heterogeneity was assessed using the I² test and yielded a value of 48%, indicating moderate heterogeneity. This variation may be attributed to differences in sample sizes, follow-up durations, and surgical protocols employed across the studies.

Risk of Bias Assessment

The risk of bias assessment for the included studies revealed a range of methodological rigor, with NOS scores varying from 4 to 9 out of a maximum of 9 points. Studies 1, 3, 6, and 11 demonstrated robust methodologies with high scores (8-9), reflecting comprehensive selection criteria, adequate adjustment for confounders, and reliable outcome assessments. As these studies include randomized controlled trials or prospective designs with sufficiently large sample sizes, their findings are highly reliable.

In contrast, study 4 (2019) and study 5 (2023) scored lower at 4/9 and 6/9, respectively. This was due to limitations such as small sample sizes, lack of adjustment for confounding factors, and shorter follow-up durations. Additionally, case reports and single-center studies generally scored lower, reflecting concerns about their representativeness and generalizability. Despite these limitations, the findings of these studies can still provide valuable context, albeit with caution due to their higher risk of bias.

Discussion

The SST has gained increasing attention in implant dentistry due to its unique ability to preserve the buccal bone plate and soft tissue structures during implant placement. This systematic review synthesizes findings from various studies to evaluate the SST's survival outcomes, limitations, and future directions, providing practical insights into its clinical application.

The findings of this review emphasize the ability of the SST to improve implant survival rates by maintaining alveolar ridge integrity. Multiple studies have demonstrated that the SST significantly reduces horizontal and vertical bone resorption compared to conventional implant placement. Gluckman et al. (2016) reported a reduction in buccal bone resorption in SST-treated patients over a three-year follow-up period, with over 95% of implants achieving successful integration and maintaining stable peri-implant tissue health [24].

The SST also demonstrates superior aesthetic outcomes, particularly in high-demand regions such as the maxillary anterior. Venkatraman et al. (2023) conducted a randomized controlled trial comparing the SST to the conventional technique in immediate implant placements. The study demonstrated that the SST resulted in a significantly higher PES and reduced buccal soft tissue volumetric changes, indicating superior peri-implant tissue aesthetics. These findings support the effectiveness of the SST in preserving the periodontal ligament and bundle bone complex, which is essential for achieving natural-looking outcomes [25].

Despite these promising outcomes, the survival rates are not universal. A systematic review and meta-analysis by Lin et al. (2022) evaluated the clinical feasibility of the SST in immediate implant placement. The study found that the SST resulted in lower marginal bone loss and higher PES compared to a conventional immediate implant placement. However, the authors cautioned against routine clinical implementation of the SST due to the lack of large-scale randomized controlled trials and long-term follow-up data. This underscores the importance of careful patient selection and risk assessment when considering this technique, particularly in individuals with systemic conditions or habits that may compromise healing and implant stability, like smoking [26].

While the SST has significant advantages, it has its limitations. One of the primary challenges identified in this review is the high level of technical skill required to successfully perform the procedure. To retain the buccal shield in the SST, meticulous preparation is needed to prevent complications such as shield mobility, exposure, or fracture. Gluckman et al. (2018) reported that internal exposure of the socket shield was the most frequently observed complication, and this is often caused by operator inexperience or suboptimal surgical execution [27].

Other complications, including soft tissue recession and peri-implantitis, have also been documented. A case report by Godil et al. (2022) discussed an SST-associated complication in a patient with a thin gingival biotype. The patient experienced soft-tissue recession that led to shield exposure, and this was attributed to the thin gingival biotype and insufficient soft-tissue thickness. The authors emphasized the importance of careful patient selection and suggested that performing a connective tissue graft at the time of implant placement can minimize or eliminate potential soft-tissue complications in these situations [28].

Another limitation is the lack of robust, long-term data assessing SST outcomes beyond five years. Most of the studies included in this review focused on short- to mid-term outcomes, leaving questions about the technique’s durability unanswered. A systematic review by Alhachache et al. (2022) emphasized the necessity for additional long-term studies that assess the SST’s efficacy and prognosis. The authors concluded that while the SST is a promising method for preventing post-extraction ridge alterations and preserving esthetics, its highly sensitive surgical protocol necessitates a skilled surgeon [29].

To address the challenges identified in this review, several strategies can be used. Advances in digital dentistry, such as CAD/CAM systems, hold significant potential in improving the precision of SST procedures. For instance, Chen et al. (2020) introduced a digital approach utilizing a computer-aided design and CAD-CAM titanium preparation template specifically for the SST. This method aims to standardize the surgical protocol, thereby minimizing operator variability and enhancing the reproducibility of SST outcomes [30].

Biomaterials that promote soft tissue healing and reduce the risk of peri-implant complications are another promising area of development. Bioactive coatings and regenerative scaffolds have been shown to enhance peri-implant tissue integration, mitigating risks associated with microbial colonization or soft tissue recession [31].

Furthermore, educational programs and specialized training in the SST are essential to reduce operator-dependent variability and improve clinical outcomes. Comprehensive training will ensure that clinicians develop the skills needed to perform the procedure accurately and address complications effectively.

Future research should include long-term, multicenter RCTs that examine the SST’s performance across diverse patient populations and clinical scenarios. These studies should evaluate the impact of systemic health conditions, such as diabetes or smoking, on SST outcomes and establish standardized guidelines for case selection and surgical execution.

Limitations and Future Directions

While this review offers valuable insights into the clinical outcomes and complications associated with the SST, several limitations must be acknowledged. First, the heterogeneity of included studies in terms of design, surgical protocols, and follow-up periods limits the ability to perform a uniform quantitative synthesis. Second, many studies were small in scale, lacked randomization, or were case series with inherent bias risks, affecting the generalizability of the results. Third, the absence of standardized outcome measures - especially regarding aesthetic assessment and patient-reported outcomes - introduces variability in interpretation.

Additionally, most studies had short- to medium-term follow-up durations, with limited data beyond five years. The influence of operator experience, case selection criteria, and adjunctive therapies such as grafts or digital guidance was also inconsistently reported.

Future research should prioritize well-designed, multicenter randomized controlled trials with long-term follow-up to validate the survival and aesthetic benefits of the SST. Standardizing surgical protocols and outcome measures, incorporating digital technologies, and evaluating patient satisfaction and quality of life will be essential to enhance the clinical adoption of the SST.

## Conclusions

The SST represents a significant advancement in implant dentistry, offering superior preservation of peri-implant tissues and aesthetic outcomes compared to traditional methods. However, its adoption is limited by technical sensitivity, procedural challenges, and a lack of robust long-term data. Current evidence underscores the importance of patient selection and meticulous surgical execution in achieving optimal results. To realize its full potential, concerted efforts are needed to address the SST’s limitations through innovative research, technological advancements, and standardized training protocols. By addressing these issues, the SST could emerge as a mainstream technique that aligns clinical outcomes with the growing demand for minimally invasive and aesthetically focused implant solutions.

## References

[1] Schropp L, Wenzel A, Kostopoulos L, Karring T (2003). Bone healing and soft tissue contour changes following single-tooth extraction: a clinical and radiographic 12-month prospective study. Int J Periodontics Restorative Dent.

[2] Gluckman H, Du Toit J, Salama M (2016). The pontic-shield: partial extraction therapy for ridge preservation and pontic site development. Int J Periodontics Restorative Dent.

[3] Araújo MG, Lindhe J (2005). Dimensional ridge alterations following tooth extraction. An experimental study in the dog. J Clin Periodontol.

[4] Botticelli D, Berglundh T, Lindhe J (2004). Hard-tissue alterations following immediate implant placement in extraction sites. J Clin Periodontol.

[5] Kan JY, Rungcharassaeng K, Lozada J (2003). Immediate placement and provisionalization of maxillary anterior single implants: 1-year prospective study. Int J Oral Maxillofac Implants.

[6] Hürzeler MB, Zuhr O, Schupbach P, Rebele SF, Emmanouilidis N, Fickl S (2010). The socket-shield technique: a proof-of-principle report. J Clin Periodontol.

[7] Blaschke C, Schwass DR (2020). The socket-shield technique: a critical literature review. Int J Implant Dent.

[8] Bäumer D, Zuhr O, Rebele S, Hürzeler M (2017). Socket Shield Technique for immediate implant placement - clinical, radiographic and volumetric data after 5 years. Clin Oral Implants Res.

[9] Oliva S, Capogreco M, Murmura G, Lupi E, Mariachiara DC, D'Amario M (2023). The socket shield technique and its complications, implant survival rate, and clinical outcomes: a systematic review. J Periodontal Implant Sci.

[10] Mujawar S, Devkar N, Vibhute A, Deshpande M, Mali P (2018). Socket shield technique: a review. Int J Recent Sci Res.

[11] Coiffic S, Soulas H, Hamon J (2024). Immediate extraction-implantation and provisionalization (IIP): esthetic evaluation of IIP surgical protocols via the Pink Esthetic Score: a retrospective cross-sectional study of 39 implants. J Oral Med Oral Surg.

[12] Gharpure AS, Bhatavadekar NB (2017). Current evidence on the socket-shield technique: a systematic review. J Oral Implantol.

[13] Atef M, El Barbary A, Dahrous MS, Zahran AF (2021). Comparison of the soft and hard peri-implant tissue dimensional changes around single immediate implants in the esthetic zone with socket shield technique versus using xenograft: a randomized controlled clinical trial. Clin Implant Dent Relat Res.

[14] Esteve-Pardo G, Esteve-Colomina L (2023). Clinical and radiographic evaluation of OsseoSpeed EV implants. Int J Oral Maxillofac Implants.

[15] Gómez-Meda R, Rizo-Gorrita M, Serrera-Figallo MA, Esquivel J, Herraez-Galindo C, Torres-Lagares D (2022). Dimensional changes in the alveolus after a combination of immediate postextraction implant and connective grafting and/or socket shield technique. Int J Environ Res Public Health.

[16] Habashneh RA, Walid MA, Abualteen T, Abukar M (2019). Socket-shield technique and immediate implant placement for ridge preservation: case report series with 1-year follow-up. J Contemp Dent Pract.

[17] Pohl S (2024). Effects of socket-shield therapy on inter-implant papilla preservation between upper central and lateral incisors: a case series with 3-5 year follow-up. J Esthet Restor Dent.

[18] Santhanakrishnan M, Subramanian V, Ramesh N, Kamaleeshwari R (2021). Radiographic and esthetic evaluation following immediate implant placement with or without socket shield and delayed implant placement following socket preservation in the maxillary esthetic region - a randomized controlled clinical trial. Clin Cosmet Investig Dent.

[19] Santhanakrishnan M, Subramanian V, Arul D, Marimuthu SV (2024). Evaluation of timing of implant placement in maxillary esthetic zone with type I extraction sockets- a randomized controlled trial. Clin Cosmet Investig Dent.

[20] Shadid RM (2022). Comparing dual-zone immediate implant placement and socket shield technique for ridge width changes in the maxilla: a prospective cohort study. Clin Cosmet Investig Dent.

[21] Shadid RM (2022). Socket shield technique and delayed implant placement in maxilla: a series of five case reports. BMC Oral Health.

[22] Shadid RM (2022). Immediate implant placement with socket shield technique in the maxilla: a prospective case series evaluation at 1-year follow-up. Head Face Med.

[23] Sun C, Zhao J, Liu Z, Tan L, Huang Y, Zhao L, Tao H (2020). Comparing conventional flap-less immediate implantation and socket-shield technique for esthetic and clinical outcomes: a randomized clinical study. Clin Oral Implants Res.

[24] Gluckman H, Salama M, Du Toit J (2016). Partial extraction therapies (PET) part 1: maintaining alveolar ridge contour at pontic and immediate implant sites. Int J Periodontics Restorative Dent.

[25] Venkatraman N, Jain V, Nanda A, Koli DK (2023). Comparison of soft tissue volumetric changes and pink esthetics after immediate implant placement with socket shield and conventional techniques: a randomized controlled trial. Int J Prosthodont.

[26] Lin X, Gao Y, Ding X, Zheng X (2022). Socket shield technique: a systemic review and meta-analysis. J Prosthodont Res.

[27] Gluckman H, Salama M, Du Toit J (2018). A retrospective evaluation of 128 socket-shield cases in the esthetic zone and posterior sites: partial extraction therapy with up to 4 years follow-up. Clin Implant Dent Relat Res.

[28] Godil AZ, Kazi AI, Hegde R, Lambe S, Kheur M (2022). Management of a complication with partial extraction therapy: a clinical case letter and clinical recommendations. J Oral Implantol.

[29] AlHachache S, Husseini B, Younes R (2022). Socket-shield technique: where do we stand today?. J Osseointegration.

[30] Chen L, Yang Z, Liu X, Lin WS, Tan J (2020). CAD-CAM titanium preparation template for the socket-shield technique. J Prosthet Dent.

[31] de Avila ED, van Oirschot BA, van den Beucken JJ (2020). Biomaterial-based possibilities for managing peri-implantitis. J Periodontal Res.

